# The Benefit of an Umbrella Protocol: Reducing Challenges in Orthopedic Oncology Research

**DOI:** 10.3390/jcm13061551

**Published:** 2024-03-08

**Authors:** Samuel K. Simister, Shannon Tse, Aziz Saade, Chancey A. Sweeney, Barton L. Wise, Steven W. Thorpe, R. Lor Randall

**Affiliations:** 1 Department of Orthopaedics, University of California, Davis, CA 95817, USA; sksimister@ucdavis.edu (S.K.S.);; 2 Department of Internal Medicine, University of California, Davis, CA 95817, USA

**Keywords:** orthopedic oncology, oncologic research, rare disease research, research challenges

## Abstract

**Background**: Orthopedic oncology research is hindered by the scarcity of musculoskeletal tumors and research administrative inefficiencies. This paper introduces observational research through an innovative institution-specific methodology—termed an umbrella protocol. This protocol outlines a comprehensive standard procedure to expedite ethical approval for future aligned studies, reducing administrative barriers to research. **Methods**: We developed an umbrella protocol at an academic center, involving meticulous methodological identification and coordination with the institutional review board (IRB) to adhere to local guidelines. The protocol encompasses identifying investigators, research objectives, study goals, and data and safety monitoring frameworks necessary for typical standards. **Results**: Implementation of the umbrella protocol took 110 days to achieve exemption status, following multiple discussions with the IRB and extensive revisions. At the authors institution, this protocol significantly reduces protocol review times from an average of six-to-eight weeks to nearly instantaneous, facilitating a streamlined research process. Additionally, we established a dedicated orthopedic oncology patient registry to enhance future research endeavors. **Conclusions**: The adoption of umbrella protocols represents a pioneering strategy in orthopedic oncology. This approach mitigates research administrative burdens and broadens research scope in the field. It underscores the necessity of IRB collaboration, methodological precision, and stringent data management. The article also reflects on the ethical implications and potential biases introduced by emerging technologies like artificial intelligence, advocating for diligent ethical oversight. The establishment of an umbrella protocol marks a significant step towards more efficient research methodologies, ultimately aiming to improve patient care and outcomes for individuals with rare musculoskeletal conditions.

## 1. Introduction

### 1.1. The Challenges of Research in Orthopedic Oncology

Musculoskeletal (MSK) oncology is a distinct specialty encompassing the complexities of primary and metastatic bone cancer, soft tissue sarcoma, and benign bone tumors, compounded by the potential for significant treatment and disease-associated morbidity. In recent years, MSK oncology has dramatically evolved, focusing on enhancing patient care through advancements in multimodal management strategies, surgical techniques, medical and pharmacological treatments, patient-reported outcomes, and surveillance protocols [1,2]. While continuing to evolve, the pathologies in MSK oncology pose many challenges in developing clinical evidence for informed decision-making due to the complex manifestations and heterogeneous disease sequelae and their rarity [3]. This is partially because MSK and soft tissue tumors exhibit significant diversity, encompassing multiple subtypes with distinct histology and genomics. This heterogeneity poses difficulties in classification, understanding the underlying biology, and developing targeted treatments or decision-making protocols [4].

As proponents of evidence-based medicine call for higher quality studies in orthopedic oncology [5,6], it is important to consider the rarity and complexity of cases that may make clinical trial methodologies impractical or ethically questionable. Additionally, the rarity of bone and soft tissue tumors presents challenges in recruiting study participants, especially in the pediatric or adolescent population, and limits the availability of specimens for research and conducting larger-scale trials [4]. Examining the long-term outcomes of orthopedic oncology treatments necessitates extended follow-up periods, which is challenging because research in this field often receives less funding than more prevalent cancers due to their rarity [7]. This increases the challenges of obtaining significant findings due to the limited number of patients and the time required for outcomes to manifest. Furthermore, the difficulty in early-stage detection of MSK tumors and late patient presentation due to non-specific symptoms can result in delayed diagnosis and treatment initiation, impeding the exploration of early disease stages and the development of effective preventative measures [8].

Despite these challenges, ongoing research efforts strive to advance comprehension of MSK oncology, improve early detection methods, and devise more effective treatments for patients, which is often accomplished via observational studies. Hoppe et al. discussed the value of observational studies in an article published in 2009 that outlined specific considerations of surgical treatment groups, sample sizes, and randomization that make random or non-randomized trials sub-optimal [9]. They added specifics on the benefits of observational studies with good inclusion criteria to address “*Questions concerning etiology, prognosis, and estimates of potential risk or harms of treatment*”, which, when combined with controlled trials, may “*produce a more complete picture of the potential benefits and harms of a clinical decision for individual patients or health systems*” [10]. Thus, to enhance clinical decision-making, efforts in observational research require efficient methods of data collection to provide substantial evidence, highlighting the need for streamlined and efficient research processes, including navigating the requirements of one’s local Institutional Review Board (IRB), essential to improving orthopedic oncology research.

### 1.2. A Brief History of the Institutional Review Board

The history of the IRB dates to the 1960s, when the National Institutes of Health (NIH) proposed impartial peer review for all human subjects’ research [11]. Governed by FDA regulations, an IRB oversees and monitors clinical research involving human participants, ensuring the implementation of ethical guidelines to protect study participants [12]. The scope and responsibilities of IRBs have evolved significantly, especially after the 2012 and later revisions to the Common Rule, which opened new avenues for expedited research protocols for non-exempt human research [13]. This includes studies deemed to pose little to no risk to patients, such as observational chart reviews.

While the IRB plays a crucial role in clinical research, its drawbacks include cumbersome paperwork, time delays, and other administrative inefficiencies. Given that the typical cadence for IRB panel submission review is monthly, this may lead to delays in processing clinical research protocol proposals [14]. Recent literature has explored surgeon challenges in managing relationships and administrative requirements to complete an IRB [15]. Articles have reported greater than one month of wait time for exempt studies and a three-to-four-month average wait time for others [16,17,18]. In contrast, other articles have discussed decreased participation or data collection by those requiring more in-depth local IRB approval [19]. Additionally, in the author’s experience, it is not uncommon to spend months revising oncologic protocols only to find that, after receiving data reports, sample sizes for accessible data are inadequate to produce meaningful research.

This historical context underscores the need for more efficient administrative research processes, particularly in orthopedic oncology, where time-sensitive and impactful research is crucial because of sampling and vulnerable populations.

### 1.3. Improving the Efficiency of Orthopedic Oncology Research

This article explores the promising strategy of establishing an umbrella protocol, which is a singular methodology that can be approved via the local IRB to cover multiple observational studies, thus drastically reducing the number of IRB approvals needed for observational research and increasing evidence for the treatment of rare diseases. We share a specific example of an approved umbrella protocol at our institution and discuss considerations for the future of rare disease research, including how artificial intelligence (AI) and machine learning (ML) may impact future research. By discussing how to properly execute an umbrella protocol, our objective is to outline a cohesive and standardized approach to guiding research initiatives in orthopedic oncology so that others may bolster research efficiency by increasing capacity and directing effort toward core study activities, ultimately enabling a more robust understanding of treatment outcomes and effectiveness for orthopedic oncologic disease.

## 2. Methods

### 2.1. What Is an Umbrella Protocol?

An umbrella protocol is a comprehensive research protocol that specifies scholarly processes that allow for additional novel study hypotheses and projects to be grandfathered into previously established IRB approvals if they follow similar procedures. Umbrella protocols allow a single submission to cover a multitude of research endeavors, some of which may not yet be considered. Through this process, an umbrella protocol can directly address some of the historical challenges of administrative research activities, including removing the need for drafting, submitting, revising, and processing additional protocols for similar, pre-defined study types. An example of these conditions is seen in Table 1.

By understanding the historical context or research oversight and some of the specific applications of an umbrella protocol, surgeon-scientists can establish a strategy to create methods that suit their needs. The following section will detail some recommendations on how to approach this process.

### 2.2. Establishing an Umbrella Protocol

#### 2.2.1. Step One: Local Administration

Since the IRB process is highly variable across institutions, initiating an umbrella begins with a better understanding of local policies [5]. A researcher should start by exploring research guidelines and processes as directed by their institution and adhere to these early to streamline the approval process for the research protocol.

Once familiar with the territory, a successful umbrella protocol requires early and regular meetings with IRB staff and leadership [5]. The process of bridging administrative and active aspects of research is improved with open communication, persistence, and patience in an engaged and collaborative atmosphere between IRB staff and the proposing unit. Researchers and administrators can find standard solutions and avoid unnecessary pitfalls by defining goals early. Part of this discussion includes safety and monitoring details and compliance to mitigate the confidentiality risks related to retrospective studies [16,20]. In the authors’ experience, this must be established both in partnership with the IRB and internally within the clinical research administration of the proposing department.

#### 2.2.2. Step Two: Major Components

After identifying local processes and forming a collaborative partnership with local research boards, the second step in generating an umbrella protocol includes the following major components as exemplified in Table 2:

Investigators: Determining the primary, co-, and other investigators is the first significant component of generating an umbrella protocol. This includes special consideration for the responsibilities of the primary investigator (PI) and the department responsible for the integrity of all projects under the umbrella. This can be accomplished by meticulously adhering to internal protocols, including monitoring participating investigators for the appropriate research qualifications and training.

Research processes: The next essential parameter to generating an umbrella protocol includes creating a specific and clear methodology. These parameters should dictate the specific research processes repeated for each study that may fall under the protocol. These may include type of study, location, inclusion and exclusion criteria, study variables, data mining, analysis, and patient interaction. The procedures must be clearly defined in the protocol and adhered to meticulously to maintain high standards of research, which, over time, may require continued iteration and revision with the IRB. This is a crucial step that will specifically detail how future studies may avoid the common biases that are common within observational research [21], and, for this, the authors also recommend adhering to policies outlined in the STROBE (Strengthening the Reporting of Observational Studies in Epidemiology) checklist [22].

Ethical considerations: Perhaps one of the most critical components in the development of the umbrella protocol is incorporating processes that protect data and safety according to traditional research ethics. For data management and confidentiality, carefully identifying what protected health information (PHI) is at risk allows the researcher to detail this information for the IRB (Table 3). Data management should also include specifics on how identifier keys will be used, how long data will be stored, and methods of destroying expired data. Although not specific to PHI, additional data elements from potential sources should be considered and catalogued.

#### 2.2.3. Step Three: Protocol Submission

The final step in creating an umbrella protocol includes the process of submitting and revising the protocol via the collaborative partnership generated in step one. This process will identify the specific details required by local administration to ensure appropriate research methods and procedures; patient population and PHI; data collection, management, and confidentiality; access and reporting; as well as any unique inclusions based on special considerations. This is an iterative process that varies in need and timing for every institution.

### 2.3. Special Considerations

Local registry: One consideration for improving the reach of an umbrella protocol includes the creation of an institutional registry of MSK oncology patients that allows for basic information to be categorized in a standardized fashion for quickly identifying diagnoses, medical treatments, and other interventions. This registry can be created from existing daily or weekly triage lists or created anew. Specific variables to be considered may include medical record numbers, age, diagnosis, medications, orthopedic interventions, adjuvant therapies, and oncologic outcomes.

National registries: Researchers may expand upon local data by exploring additional information from national registries (see Table 4). Notably, while some institutions may not require research approval when using public data, ethical research practices recommend applying to one’s local IRB to be granted exempt status.

Global perspectives: Multi-center international collaboration can speed up research advancements by allowing socioeconomic diversity and cross-cultural interactions. This is particularly beneficial in less prevalent diseases, as in the case of several orthopedic oncology pathologies. Yet, one of the many barriers to overcome in such collaborations, besides establishing data use agreements, is the ethical regulations that can vary between and within countries [23,24]. Besides, using central or federated IRBs, umbrella protocols can partly contribute to designing a uniform framework and speed up ethical approval in these global collaborations [5]. An important key step is understanding the nuances and managing the variations in the ethical committees and regulations across the different nations [25].

In the European Union, IRBs are also known as independent ethics committees or research ethics committees [26]. The European Network of Research Ethics Committee emphasizes establishing the infrastructure to harmonize and centralize committees, thus enhancing knowledge sharing. In Canada, research ethics boards are the equivalent of IRBs, regulated by the Tri-Council policy statement for research involving humans [27,28].

While regulations vary between countries, the PI, regardless of its institutional location, must adhere to the international standards of ethical research conduct and provide equivalent protections to human subjects in foreign countries [29,30]. Furthermore, the PI must consider the different authority’s regulations even when collaborating with a remote campus within the same institution [31,32].

With a comprehensive understanding of the special considerations and major components, an umbrella protocol has the potential to harmonize a specific type of research method and procedure, which has the potential to increase local and global research efficiencies while, at the same time, maintaining high ethical standards of conduct in the research. In the following section, we will share some of the methodologies of the active umbrella protocol at our institution.

## 3. Results

### 3.1. Outcomes of Our Umbrella Protocol

From start to finish, our completed umbrella protocol required 110 days to be determined exempt, including significant contributions from a postdoctoral researcher, weekly research team meetings, interdepartmental discussions, four separate meetings with IRB personnel, and three major revisions. Importantly, it is supported by an internal clinical research team of employees within the Department of Orthopedics that oversees data safety and monitoring to ensure the appropriate ethical standards are maintained.

While relatively new at our current institution, the effectiveness of our current umbrella protocol has led to the immediate initiation of six new studies within our oncology service, where our current IRB time-to-approval averages over six weeks. Additionally, the improved relationship with our department and the local IRB is now leading to the development of a department-wide umbrella protocol for all observational research studies to include all orthopedic subspecialties.

### 3.2. Major Components

At our institution, the chief of the Sarcoma Service is primarily responsible for the approved umbrella and serves as PI. The investigative team includes an additional tumor surgeon, fellow, full-time researchers, and medical students. The procedures under our umbrella protocol are structured into three primary segments: the patient registry, research processes, and analysis and reporting.

Our MSK Oncology Registry is a foundational element for recording diagnoses and interventions stored and maintained in the Research Electronic Data Capture (REDCap) software v14.0.15. After raising a clinical inquiry, scholarly exploration of the registry allows for identifying specific cohorts within the database for preliminary analysis. If acceptable, this progresses to collect additional patient data via observational chart review, including analysis via common statistical evaluation supported by the Clinical and Translational Science Center.

Participant criteria for the umbrella protocol are inclusive, covering patients of all ages who present to the orthopedic oncology team to evaluate and manage primary and secondary cancers and tumors of the musculoskeletal system, including metastatic bone disease. No exclusions are based on personal demographics, ensuring a wide-ranging and comprehensive patient pool. For the present study, the following scientific hypothesis was created to describe the aims of our umbrella protocol:

“*Hypothesis: Over time, this umbrella protocol will be inclusive of all studies that fall within retrospective reviews of orthopedic and MSK oncology patients, including future studies that have not yet been determined but will be based on clinical questions as determined by attendings within the orthopedic oncology service. All hypotheses to be examined will be observational and include clinical questions on outcomes within orthopedic and MSK oncology. These include operative vs. non-operative management, the efficacy of commonly used orthopedic devices and biologics, and the cost-effectiveness of orthopedic oncologic management and care*”.

Ethical considerations are paramount in our protocol. Access to the registry and observational studies requires standard CITI training as mandated by the home institution. A detailed personnel list maintained by the PI and Clinical Research Coordinator ensures that all research participants meet these requirements. The collected identifiers include sensitive PHI, specific name, medical record number, date of birth, demographics, and orthopedic-related details. A comprehensive list of all variables identified for our umbrella is shown in Table 5. The registry’s lifespan is tied to the activity of the IRB, and specific studies within the umbrella protocol will manage data according to the guidelines. When completed, data from each study are destroyed within a year of the final report or if deemed non-significant in research terms. Regular updates to the IRB for event monitoring, annual reporting, and protocol modifications are managed on an ad hoc basis, ensuring continuous oversight and ethical compliance.

## 4. Discussion

Research in orthopedic oncology has faced many challenges historically, making it difficult to gather the data needed for prospective trials or to impact decision-making [33,34]. As quality data becomes increasingly pertinent, the creation of an umbrella protocol and an accompanying registry can relieve a great deal of the start-up effort needed for observational studies. Additionally, the improved efficiency can facilitate obtaining funding for orthopedic oncology research while also decreasing the challenges of prospective work [35]. The present study discusses several considerations for generating an institutional umbrella protocol to promote research efficacy, making it the first article in the literature to detail the approach to establishing an umbrella protocol.

The implementation of an umbrella protocol at our institution marks a significant stride in optimizing research efficiency. By consolidating multiple research endeavors under a single protocol, we have observed a noticeable reduction in the time and resources traditionally expended in the IRB approval process. This streamlined approach contrasts starkly with the prolonged and often repetitive nature of conventional IRB submissions, including the severe variability in accepted methods [15], or timing [16], that can exist between institutions, illustrating the protocol’s efficacy in expediting research initiatives. Beyond administrative convenience, these protocols enhance the quality of patient-oriented research for populations of rare diseases by creating an institutional registry that, via private or multi-center review boards, may have a significant future impact on aggregating outcomes. Additionally, the ability to quickly identify and analyze cohorts of rare patient populations within these registries enhances the availability of data, which may only be improved with future advancements in AI. Thus, these registries may prove invaluable for answering critical clinical questions about the outcomes and cost-effectiveness of both surgical and non-operative interventions.

Another aspect of improving the quality of research within orthopedic oncology may include consideration of how AI-generated data and analysis may work within the scope of an umbrella protocol to enhance the quality and scope of research. Recent AI applications within the world of orthopedic surgery include disease and outcome prediction, medical image analysis, and decision support systems, which present methods for the analysis of large amounts of data that prove too tedious for current research techniques [36]. Opportunities from big data, including continually improving databases and AI, may underline a more significant benefit to protocols that bring novel insights and efficiencies previously unattainable. By incorporating AI and ML into registry-based research, these technologies would excel in querying and analyzing vast quantities of data that can be too complex when using traditional methods, including sifting swiftly through large datasets and identifying patterns and predictive models that could be invaluable in rare diseases.

Despite this potential, the evolution of AI and ML raises concerns regarding the privacy of patient information and inherent biases within algorithmic learning models. The ability of AI to accurately generate predictive analyses of patient data is dependent on large amounts of high-quality patient information, which is generally stored on the networks of medical institutions, often lacking secure connections to AI systems [37]. These systems are largely stored and managed by private corporations, which have a history of poor protection of privacy, leading some to call for greater systematic oversight of big data health research [38]. The consideration of data privacy is even more concerning when considering the large amount of data generated from personal devices that leads to data exploitation, tracking, and biometric recognition [39]. Recent studies have highlighted how AI and ML can use these data to reidentify patient identities even after the data has been scrubbed of PHI [38]. Beyond privacy concerns, training AI for clinical research has been shown to present certain types of errors and biases that are difficult to supervise [38]. One unique aspect of this is termed the ‘black box’ problem, which describes the methods and reasoning AI-conclusions that are uninterpretable to human observers. Another includes the consideration of datasets utilized for training AI algorithms, which may render ML models nongeneralizable to all populations or may generate clinical decision errors when presented with unexpected contexts [36]. Thus, while these examples detail the need for increased ethical research governance and ethical oversight for practices and confidentiality, in the setting of orthopedic oncology, the potential evolution of sophisticated AI analyses may offer greater opportunities to improve the quality of research and decision-making within rare disease research.

### Limitations

The generation of an umbrella protocol has some limitations. First, implementing such protocols may not be generalizable; they depend on the infrastructure and resources of individual institutions, and those with limited resources may face challenges in establishing and maintaining comprehensive registries and databases required for effective umbrella protocol operation. Second, relying on retrospective data, a common element in umbrella protocols, may introduce biases inherent to retrospective analyses. Additionally, the generalizability of findings may be limited by the specific demographic and disease characteristics of the patient population at the home institution. Thus, it may not be representative of all patient populations. Third, while umbrella protocols aim to streamline research processes, they may inadvertently lead to a one-size-fits-all approach that overlooks the special nuances of individual studies. This can potentially impact the depth and specificity of research outcomes. Lastly, the integration of AI and ML technologies, though promising, brings its own set of challenges, including algorithmic bias, ethical practices, and the need for continuous model validation of these novel tools in clinical settings. Future studies may address these limitations by exploring: (1) the inclusion of additional disciplines to better identify patients’ needs, (2) the establishment of standards to protect patient privacy, (3) a centralized harmonization of acceptable research practices to reduce variability, (4) multi-center registries that encourage national or international collaboration, and (5) regulation to mitigate the biases of the evolving AI and ML technologies.

## 5. Conclusions

The implementation of an umbrella protocol presents an opportunity toward a more efficient and effective study of rare diseases, addressing administrative challenges in research and the need for expansive, quality data to improve clinical evidence for orthopedic oncologic treatments. By working closely with local review boards, researchers may consolidate a singular methodology under an umbrella protocol, which has the potential to accelerate the initiation of studies and foster the integration of enhanced research methods with evolving technologies like AI and ML. Importantly, by considering ethical implications, the umbrella method necessitates vigilant oversight of research procedures, patient data, and potential biases such as algorithmic analyses. As the field of research continues to evolve with new technologies, umbrella protocols may play a crucial role in guiding the pursuit of improved quantity and quality of clinical evidence within the realm of musculoskeletal oncology.

## Figures and Tables

**Table 1:** An example of parameters for an observational umbrella protocol.

Study types:	Observational: retrospective and prospective
Site(s):	Single institution
Criterion:	Musculoskeletal oncology patients - All demographics and ages
Intervention:	Chart review (no intervention) - Patient demographics and presentation - Disease evaluation and diagnostics - Surgical and non-surgical interventions Patient-reported outcomes and survival

**Table 2:** Steps to generating an umbrella protocol.

Step one	Local administration - Review local IRB approach - Review goals with IRB leadership - Adjust protocol as needed to meet local standards
Step two	Protocol draft components - Identify the primary and all co-investigators - Generate an overall hypothesis - Describe methods, variables, and reporting - Detail data management and ethical considerations
Step three	Protocol submission - Comply with all local policies and requirements - Revise draft and processes as requested

**Table 3:** Protected health information variables.

Medical record numbers	Certificate/license numbers
Patient names	Vehicle identifiers
Dates related to the health or identity	Device attributes or serial numbers
Email addresses, telephone, and fax	Digital identifiers
Geographical elements	Biometric elements
Health insurance	Photographs of a patient’s face
Account numbers	Other identifying numbers or codes
Social security numbers	

**Table 4:** Comparison of national databases for research.

Database	Summary	Variables
HCUP	Comprehensive overview of hospital care in the United States, including information on inpatient stays, emergency department visits, and ambulatory surgeries.	Encounter-level, clinical, and nonclinical information, including all-listed diagnoses and procedures, discharge status, patient demographics, and charges for all patients, regardless of payer (e.g., Medicare, Medicaid, private insurance, and uninsured), began in 1988.
IBM MarketScan Research Database	Healthcare policies, treatment patterns, and outcomes. Particularly valuable for examining patterns of care, treatment outcomes, and the economic aspects of orthopedic treatments and interventions.	Patient-level health data (medical, drug, and dental), productivity (workplace absence, short- and long-term disabilities, and workers’ compensation), laboratory results, health risk assessments (HRAs), hospital discharges, and electronic medical records.
National Medicare Data	Valuable for studying treatment patterns, outcomes, and costs associated with various orthopedic conditions and procedures in the elderly population.	Demographics, enrollment, claims, prescription drugs, utilization, cost, quality, performance, chronic conditions, provider, and survey data.
NCDB	A nationwide oncology outcomes database by the Commission on Cancer, American College of Surgeons, and American Cancer Society, including more than 1500 facilities.	Patient demographics, tumor characteristics, treatment information, outcomes data, facility characteristics, patterns of care, diagnostic and staging information, patient follow-up, socioeconomic, geographic, and quality of care data.
NSQIP	A program managed by the American College of Surgeons (ACS) with the primary goal of enabling hospitals to improve the quality of surgical care.	Patient demographics, preoperative risk factors, intraoperative variables, postoperative outcomes, mortality, length of stay, readmission rates, reoperation rates, anesthesia, and discharge destination data.
PearlDiver	Provides a large set of anonymized patient records from various sources, including Medicare, private insurance companies, and hospital networks	Patient demographics, clinical diagnoses and conditions, procedures, treatments, medication and pharmacy claims, utilization data, healthcare costs and reimbursements, outcomes, longitudinal patient, insurance coverage, and quality measures data.
SEER-CAHPS	Combined SEER and CMS data is particularly valuable for researchers interested in the intersection of cancer epidemiology, patient experiences, and health outcomes	Cancer-specific clinical (SEER), patient experience, satisfaction, demographic, socioeconomic, healthcare utilization, costs, patient-reported outcomes, geographic, environmental, longitudinal, and insurance coverage data.

**Table 5:** Overview of variables listed in an approved umbrella protocol.

PHI	Patient names, Geographical elements, Related Dates, Medical record numbers, Health insurance,
Background	Medical comorbidities, social habits, socioeconomic, educational, and demographic status, review of systems, general health status, pain status, psychological status, musculoskeletal status, and quality of life status.
Clinical evaluation	Treatment team, HPI, Cancer specifics, year of diagnosis, grading, staging, Clinical findings, Range of Motion, Strength, Weakness, Laxity, Alignment, Effusion, Passive Motion Deficit, Ligament Exam (manual, instrumented, X-ray), Impingement, Crepitus, Instability, Girth, Tightness, Tendonitis, etc., Patient-reported outcomes (PROMIS-29, KPS, EQ-5D, TESS, etc.), Laboratory values, Diagnostics, X-ray films, CT Scan, MRI/Arthrogram, Nuclear Medicine Scans, Metastatic Bone Scan, EKGs, POCUS, etc., Medications received
Interventions	
Neoadjuvant	Cell-based and other targeted therapies, Chemotherapy, Immunotherapies, Radiation, Immunotherapy, Hormone Therapy
Operative	Date of surgery, Surgeon name, Anesthesia type, Surgery Time (skin to skin in minutes), Pre-op diagnosis, Intraoperative interventions, Anesthesia, antibiotics, blood products, growth factors. etc., Procedure, Orthopedic implants, Arthroplasty devices, cerclage wires, nails, plate, screws, sutures, etc., Grafts. Adipose, bone, cartilage, osteochondral, skin, soft tissue, etc., post-op diagnosis
Adjuvant	Bio-therapies, chemotherapies, immunotherapies, radiation
Long-term data	
Complications	Intraoperative fractures, Delayed healing, Infection, Mal- or Non-union, Hardware failure, Revision surgery
Follow-up	Dates of follow-up, Clinical findings, Range of Motion, Strength, Weakness, Laxity, Alignment, Effusion, Passive Motion Deficit, Ligament Exam (manual, instrumented, X-ray), Compartment findings, Impingement, Crepitus, Instability, Girth, Tightness, Tendonitis, etc., Patient-reported outcomes (PROMIS-29, KPS, EQ-5D, TESS etc.), Laboratory values per standard of care, Diagnostics and Imaging, healing, and union time
Survival	Disease-free survival, Disease-specific survival, Local recurrence, Overall disease, and Recurrence-free survival

PHI, Protected Health Information. HPI, history of the present illness. PROMIS, Patient Reported Outcome Measures Information System. KPS, Karnofsky Performance Scale. EQ-5D, EuroQol-5-D. TESS, Toronto Extremity Salvage Score. POCUS, point of care ultrasound.

## Data Availability

Data sharing is not applicable to this article.

## References

[1] Randall R.L., Theriault R.V., Thorpe S.W., Monument M.J. (2023). Emerging innovations and advancements in the field of musculoskeletal oncology. J. Surg. Oncol..

[2] Walker K., Simister S.K., Carr-Ascher J., Monument M.J., Thorpe S.W., Randall R.L. (2024). Emerging innovations and advancements in the treatment of extremity and truncal soft tissue sarcomas. J. Surg. Oncol..

[3] Puri A. (2014). Orthopedic Oncology—“The Challenges Ahead”. Front. Surg..

[4] Nakano K. (2022). Challenges of Systemic Therapy Investigations for Bone Sarcomas. Int. J. Mol. Sci..

[5] Marsolo K. (2012). Approaches to facilitate institutional review board approval of multicenter research studies. Med. Care.

[6] Rendon J.S., Swinton M., Bernthal N., Boffano M., Damron T., Evaniew N., Ferguson P., Serra M.G., Hettwer W., McKay P. (2017). Barriers and facilitators experienced in collaborative prospective research in orthopaedic oncology: A qualitative study. Bone Jt. Res..

[7] Kamath S.D., Kircher S.M., Benson A.B. (2019). Comparison of Cancer Burden and Nonprofit Organization Funding Reveals Disparities in Funding Across Cancer Types. J. Natl. Compr. Cancer Netw..

[8] Ferguson J.L., Turner S.P. (2018). Bone Cancer: Diagnosis and Treatment Principles. Am. Fam. Physician.

[9] Hoppe D.J., Schemitsch E.H., Morshed S., Tornetta P., Bhandari M. (2009). Hierarchy of evidence: Where observational studies fit in and why we need them. JBJS.

[10] Atkins D. (2007). Creating and synthesizing evidence with decision makers in mind: Integrating evidence from clinical trials and other study designs. Med. Care.

[11] Grady C. (2015). Institutional Review Boards. Chest.

[12] US FDA Institutional Review Boards (IRBs) and Protection of Human Subjects in Clinical Trials. https://www.fda.gov/about-fda/center-drug-evaluation-and-research-cder/institutional-review-boards-irbs-and-protection-human-subjects-clinical-trials.

[13] Office for Human Research Protections Revised Common Rule. Office for Human Research Protections. https://www.hhs.gov/ohrp/regulations-and-policy/regulations/finalized-revisions-common-rule/index.html.

[14] Hyer C.F. (2010). What is an IRB, why do we need it, and what is a private IRB?. Foot Ankle Spec..

[15] Klitzman R. (2012). From anonymity to “open doors”: IRB responses to tensions with researchers. BMC Res. Notes.

[16] Khan M.A., Barratt M.S., Krugman S.D., Serwint J.R., Dumont-Driscoll M., CORNET Investigators (2014). Variability of the institutional review board process within a national research network. Clin. Pediatr..

[17] Hall D.E., Hanusa B.H., Stone R.A., Ling B.S., Arnold R.M. (2015). Time required for institutional review board review at one Veterans Affairs medical center. JAMA Surg..

[18] Helfand B.T., Mongiu A.K., Roehrborn C.G., Donnell R.F., Bruskewitz R., Kaplan S.A., Kusek J.W., Coombs L., McVary K.T., Mist Investigators (2009). Variation in Institutional Review Board (IRB) Responses to a Standard Protocol for a Multicenter Randomized Controlled Surgical Trial. J. Urol..

[19] Finch S.A., Barkin S.L., Wasserman R.C., Dhepyasuwan N., Slora E.J., Sege R.D. (2009). Effects of local institutional review board review on participation in national practice-based research network studies. Arch. Pediatr. Adolesc. Med..

[20] Morse M.A., Califf R.M., Sugarman J. (2001). Monitoring and ensuring safety during clinical research. JAMA.

[21] Evaniew N., Nuttall J., Farrokhyar F., Bhandari M., Ghert M. (2014). What are the levels of evidence on which we base decisions for surgical management of lower extremity bone tumors?. Clin. Orthop. Relat. Res..

[22] Cuschieri S. (2019). The STROBE guidelines. Saudi J. Anaesth..

[23] Søreide K., Alderson D., Bergenfelz A., Beynon J., Connor S., Deckelbaum D.L., Dejong C.H., Earnshaw J.J., Kyamanywa P., Perez R.O. (2013). Strategies to improve clinical research in surgery through international collaboration. Lancet.

[24] Timmers M., Van Dijck J.T., Van Wijk R.P., Legrand V., Van Veen E., Maas A.I., Menon D.K., Citerio G., Stocchetti N., Kompanje E.J. (2020). How do 66 European institutional review boards approve one protocol for an international prospective observational study on traumatic brain injury? Experiences from the CENTER-TBI study. BMC Med. Ethics.

[25] Hedgecoe A., Carvalho F., Lobmayer P., Raka F. (2006). Research ethics committees in Europe: Implementing the directive, respecting diversity. J. Med. Ethics.

[26] European Network of Research Ethics Committees (EUREC) Home Page. EUREC. http://www.eurecnet.org/index.html.

[27] Health Canada About Us—Research Ethics Board. Health Canada. https://www.canada.ca/en/health-canada/services/science-research/science-advice-decision-making/research-ethics-board/about-us.html.

[28] Millum J. (2012). Canada’s new ethical guidelines for research with humans: A critique and comparison with the United States. CMAJ.

[29] University of Houston International Research. https://www.uh.edu/research/compliance/irb/policies/intl-research/.

[30] Bethesda M.D. (1978). National Commission for the Protection of Human Subjects of Biomedical and Behavioral Research.

[31] Weill Cornell, Medicine-Qatar. IRB. https://qatar-weill.cornell.edu/research/research-administration/research-compliance/human-research-protection-program/irb.

[32] Qatar, Ministry of Public Health Institutional Review Board (IRB) Registration. https://research.moph.gov.qa/en/Pages/IRB.aspx?csrt=13617144566115420196.

[33] Griggs R.C., Batshaw M., Dunkle M., Gopal-Srivastava R., Kaye E., Krischer J., Nguyen T., Paulus K., Merkel P.A. (2009). Clinical research for rare disease: Opportunities, challenges, and solutions. Mol. Genet. Metab..

[34] Ragni M.V., Moore C.G., Bias V., Key N.S., Kouides P.A., Francis C.W., Hemostasis Thrombosis Research Society (HTRS) (2012). Challenges of rare disease research: Limited patients and competing priorities. Haemophilia.

[35] Stoller J.K. (2018). The Challenge of Rare Diseases. Chest.

[36] Zsidai B., Hilkert A.S., Kaarre J., Narup E., Senorski E.H., Grassi A., Ley C., Giuseppe Longo U., Herbst E., Hirschmann M.T. (2023). A practical guide to the implementation of AI in orthopaedic research—Part 1: Opportunities in clinical application and overcoming existing challenges. J. Exp. Orthop..

[37] Yadav N., Pandey S., Gupta A., Dudani P., Gupta S., Rangarajan K. (2023). Data Privacy in Healthcare: In the Era of Artificial Intelligence. Indian Dermatol. Online J..

[38] Murdoch B. (2021). Privacy and artificial intelligence: Challenges for protecting health information in a new era. BMC Med. Ethics..

[39] Khalid N., Qayyum A., Bilal M., Al-Fuqaha A., Qadir J. (2023). Privacy-preserving artificial intelligence in healthcare: Techniques and applications. Comput. Biol. Med..

